# Notocotylid Trematodes of Wild Birds in Türkiye: A Morphological Study With New Country Records

**DOI:** 10.1002/vms3.70994

**Published:** 2026-07-08

**Authors:** Mehmet Öztürk, Şinasi Umur

**Affiliations:** ^1^ Department of Parasitology, Faculty of Veterinary Medicine Ondokuz Mayıs University Samsun Türkiye

**Keywords:** Kızılırmak Delta, notocotylus, paramonostomum, taxonomy, Waterfowl

## Abstract

**Background:**

The family Notocotylidae, established by Lühe in 1909, comprises trematode species characterized by the absence of a ventral sucker and pharynx, as well as by the presence of elongated filaments at the poles of their eggs. Members of this family primarily parasitize waterfowl, although infections have also been reported in some mammalian hosts.

**Objectives:**

The present study aimed to investigate the occurrence of notocotylid trematodes in wild birds inhabiting aquatic environments in Türkiye.

**Methods:**

A total of 57 birds representing 21 species were examined to assess the Notocotylidae trematode fauna in Türkiye. All birds were collected from the Kızılırmak Delta, a major wetland area supporting diverse avian species.

**Results and Conclusions:**

Parasitological examinations revealed the presence of *Notocotylus skrjabini*, *Paramonostomum alveoelongatum*, and *P. alveatum* in *Aythya ferina*; *Notocotylus attenuatus*, *P. alveatum*, and *P. bucephalae* in *Netta rufina*; and *N. attenuatus* in *Rallus aquaticus*. All recovered species were morphologically described, photographed, and illustrated. Notably, this study constitutes the first record of *N. skrjabini*, *P. alveoelongatum*, *P. bucephalae*, and *P. pseudoalveatum* in Türkiye.

## Graphical abstract

This study presents the first comprehensive examination of notocotylid trematodes in wild aquatic birds from the Kızılırmak Delta, Turkey. Six trematode species belonging to the genera *Notocotylus* and *Paramonostomum* were identified through detailed morphological analysis. Four species (*N. skrjabini, P. alveoelongatum, P. bucephalae*, and *P. pseudoalveatum*) are reported for the first time in Turkey, significantly expanding the known diversity and distribution of Notocotylidae in the region.

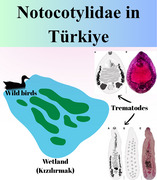

## 1 Introduction

The family Notocotylidae Lühe, 1909 comprises trematodes belonging to the order Plagiorchiida. Members of this family are commonly found in the gastrointestinal tracts of birds and mammals, where they typically inhabit the cecum. Notocotylid trematodes exhibit a relatively simple life cycle involving a single intermediate host, usually an aquatic snail. Birds become infected either by ingesting metacercariae encysted on aquatic vegetation or through the direct consumption of infected snails (Schell 1985, Jones et al. 2005).

The genus *Notocotylus* Diesing, 1839, which parasitizes birds, currently includes approximately 50 nominally described species worldwide (Diaz et al. 2020). Species of this genus are morphologically characterized by the presence of three longitudinal rows of glands on the ventral surface. In contrast, the genus *Paramonostomum* Lühe, 1909, also parasitic in birds, comprises 54 nominally described species worldwide (Bagnato et al. 2022) and is distinguished by the absence of ventral glands.

Türkiye is located within the Palearctic region and hosts a rich avifauna, with more than 500 recorded bird species, including many associated with aquatic habitats (Ramsar 1998, Karataş et al. 2021, DKMP 2022). In addition, the country contains 13 Ramsar sites that provide suitable ecological conditions for the transmission of Notocotylidae trematodes, supporting both intermediate and definitive hosts. Despite these favourable conditions, studies focusing on the diversity and distribution of notocotylid trematodes in Türkiye remain limited.

Further investigations of the family Notocotylidae are therefore essential to improve our understanding of the parasitic fauna of Türkiye. The present study aimed to document the diversity and distribution of notocotylid trematodes in wild birds from aquatic environments, with particular emphasis on identifying species previously unrecorded in the Turkish helminth fauna

## 2 Materials and Methods

### 2.1 Material Collection

In this study, a total of 57 birds representing 21 species and belonging to eight avian orders associated with aquatic habitats were examined to investigate trematode species of the family Notocotylidae. The bird species were selected based on their close association with aquatic environments and their known potential to serve as definitive hosts for notocotylid trematodes. The examined bird species and the number of individuals are presented in Table 1.

**TABLE 1:** Wild bird species examined in the present study and their sample sizes.

Order	Species	Number of birds examined	Species	Number of birds examined
Anseriformes	*Mareca strepera*	1	*Mergus albellus*	1
	*Aythya ferina* ^b,c,d^	1	*Anas platyrhynchos*	4
	*Netta rufina* ^a,c,e,f^	1		
Charadiiformes	*Scolopax rusticola*	2	*Chroicocephalus ridibundus*	1
	*Larus michahellis*	9	*Larus cachinnans*	1
Ciconiiformes	*Ciconia nigra*	1	*Ciconia ciconia*	8
Gaviiformes	*Gavia arctica*	1		
Gruiformes	*Porphyrio porphyrio*	1	*Rallus aquaticus* ^a^	1
	*Gallinula chloropus*	1		
Pelecaniformes	*Botaurus stellaris*	1	*Ardea purpurea*	1
	*Ardea alba*	1	*Egretta garzetta*	14
	*Ardea cinerea*	1	*Tachybaptus ruficollis*	1
Podicipediformes	*Podiceps cristatus*	3		
Suliformes	*Phalacrocorax carbo*	1		

^a–f^ Bird species infected with *Notocotylus attenuatus*, *N. skrjabini*, *Paramonostomum alveatum*, *P. alveoelongatum*, *P. bucephalae*, and *P. pseudoalveatum*, respectively.

Bird specimens were obtained from two sources. Some birds had died at the Department of Wild Animal Diseases, Faculty of Veterinary Medicine, Ondokuz Mayıs University, while others were found dead in the Kızılırmak Delta region, located in the Bafra District of Samsun Province. Birds collected from the Kızılırmak Delta were retrieved as soon as possible after death, generally within 24 h, to minimize post‐mortem decomposition and preserve parasite integrity.

Necropsies were performed following standard parasitological procedures. During examination, trematodes recovered from the gastrointestinal tract were rinsed in physiological saline and subsequently fixed in 70% ethanol for detailed morphological analysis. Specimens were stained with Semichon's carmine according to standard protocols and mounted in Canada balsam (Upton 2005).

Species identification was carried out using a light microscope (Nikon Eclipse 80i) by comparing morphological and morphometric characteristics with published descriptions in the relevant literature (Schell 1985, Jones et al. 2005, McDonald 1981). All examinations were conducted between 1 January, 2023, and 1 January, 2024. Sample collection and examination were performed under permit number E‐21264211‐288.04‐7092178, dated 26 September, 2022, issued by the Ministry of Agriculture and Forestry, General Directorate of National Parks.

## 3 Results

A total of 56 individual trematode specimens belonging to the family Notocotylidae were recovered from the ceca of three bird species, *Aythya ferina*, *Netta rufina*, and *Rallus aquaticus*, among the 57 birds examined, representing 21 species. Notocotylid trematodes were detected exclusively in these three host species, while no representatives of the family were found in the remaining examined birds.

The identified species and the number of collected specimens were as follows: *Notocotylus attenuatus* (one specimen from *Rallus aquaticus* and four specimens from *Netta rufina*), *Notocotylus skrjabini* (three specimens from *A. ferina*), *Paramonostomum alveatum* (35 specimens from *A. ferina* and four specimens from *N. rufina*), *P. alveoelongatum* (three specimens from *A*. *ferina*), *P*. *bucephalae* (three specimens from *N. rufina*), and *P. pseudoalveatum* (three specimens from *N. rufina*).

All recovered trematode species were subjected to detailed morphological examination and were subsequently photographed and illustrated. Of particular importance, *N*. *skrjabini*, *P*. *alveoelongatum*, *P*. *bucephalae*, and *P*. *pseudoalveatum* represent new records for the helminth fauna of Türkiye.

**FIGURE 1:**
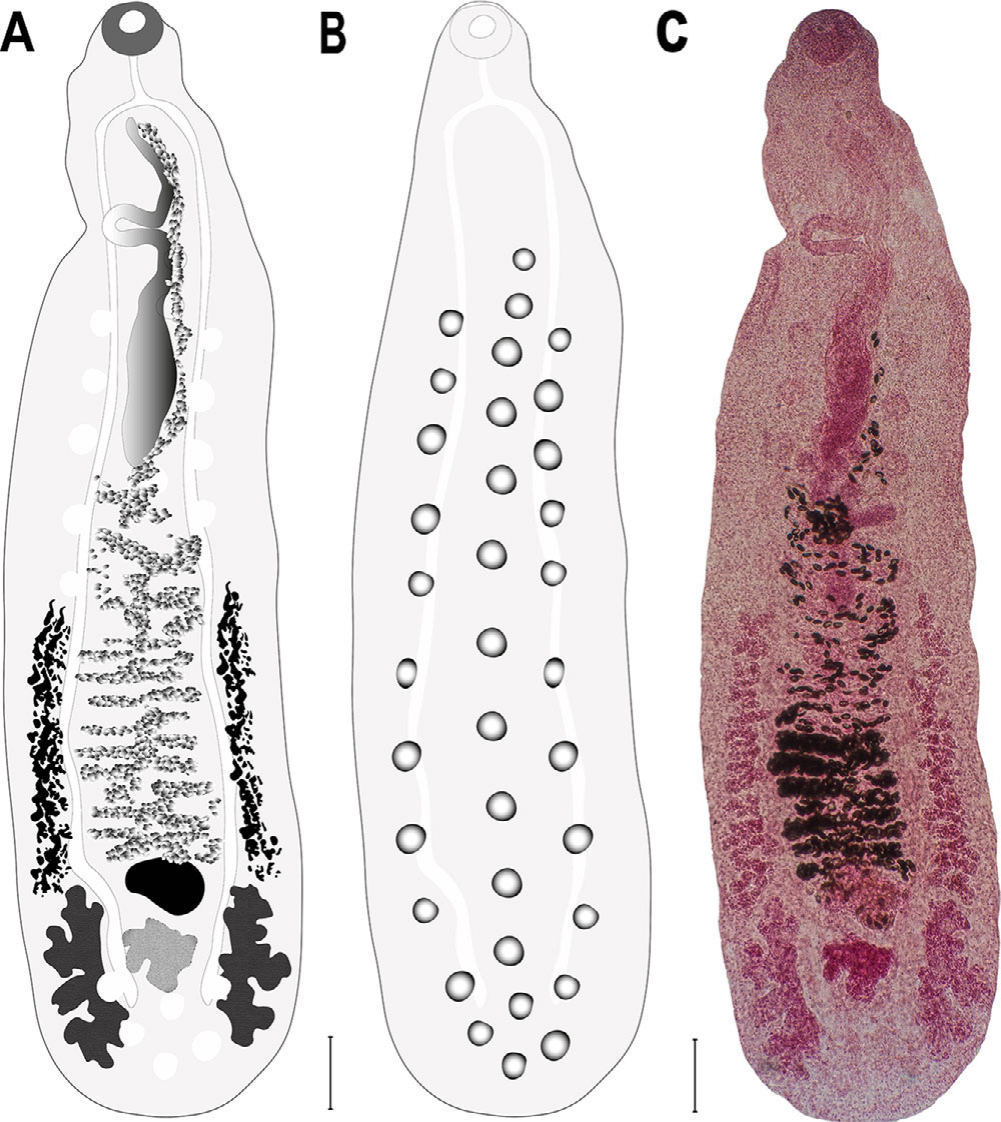
*Notocotylus attenuatus*, (A) Dorsal surface, (B) Ventral surface, (C) Light micrograph. Scale bar: 100 µm.

***Notocotylus attenuatus* Rudolphi, 1809** (Figure 1)

Host: *Rallus aquaticus*, *Netta rufina*

Site of infection: Small intestines

Intensity of infection: 1, 4

Locality: Bafra, Samsun, (41°36′N 36°05′E), Black Sea Region, Türkiye

Adults (Based on Five Specimens)

The body is elongate‐elliptical, measuring 2.0–2.2 × 0.52–0.54 mm, with a length‐to‐width ratio of 3.85. The ventral surface bears 42 ventral glands arranged in three longitudinal rows. The oral sucker is subterminal and circular, measuring 95–110 × 104–115 µm. A ventral sucker, prepharynx, and pharynx are absent.

The testes are slightly lobed and located posteriorly, situated behind the vitelline glands. The left testis measures 310–335 × 100–125 µm, while the right testis measures 464–500 × 110–115 µm. The cirrus sac is 710–750 µm in length, and the genital pore is positioned posterior to the intestinal bifurcation. The ovary is oval and slightly lobed, located between the testes in the posterior region of the body, and measures 270–280 × 110–112 µm.

The vitelline glands extend from the mid‐body to the level of the testes. The uterus is well developed and forms numerous transverse loops. Eggs measure 19–20 × 11–12 µm.

**FIGURE 2:**
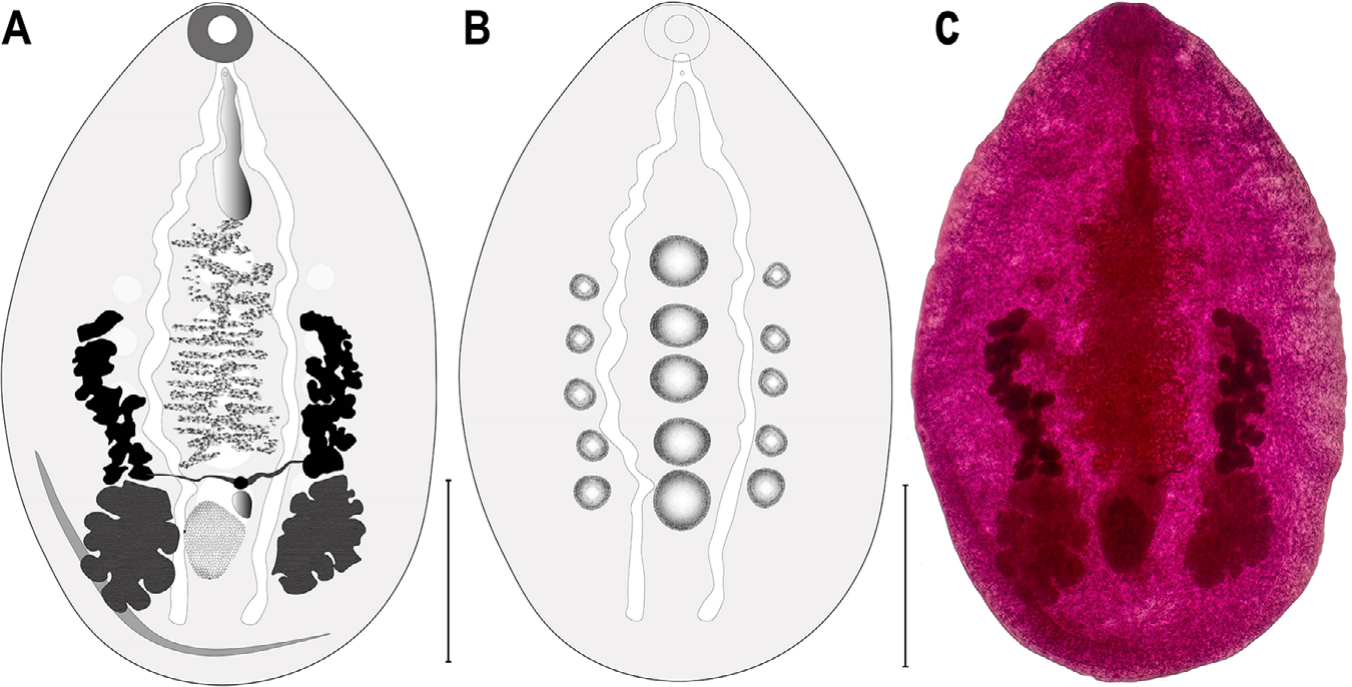
*Notocotylus skrjabini*, (A) Dorsal surface, (B) Ventral surface, (C) Light micrograph. Scale bar: 500 µm.

***Notocotylus skrjabini* Ablasov, 1953** (Figure 2)

Host: *Aythya ferina*

Site of infection: Small intestines

Intensity of infection: 3

Locality: Bafra, Samsun, (41°36′N 36°05′E), Black Sea Region, Türkiye

Adults (based on three specimens):

The body is oval to rounded, measuring 1.7–1.8 mm in length and 1.1–1.15 mm in width, with a length‐to‐width ratio of 1.54. The ventral surface bears three longitudinal rows of glands, each row consisting of five glands. The oral sucker is subterminal, circular, and measures 149–155 × 172–175 µm. A ventral sucker, prepharynx, and pharynx are absent.

The testes are slightly lobed and located in the posterior part of the body, posterior to the vitelline glands. The left testis measures 327–330 × 235–240 µm, whereas the right testis measures 328–330 × 217–216 µm. The cirrus measures 328–330 µm in length, and the genital pore is situated anterior to the bifurcation of the cecum.

The ovary is oval and slightly lobed, located between the testes in the posterior region of the body, and measures 195–200 × 139–142 µm. The vitelline glands begin at the mid‐body level and extend posteriorly to the level of the testes. The uterus is prominent and forms numerous uterine loops. Eggs measure 19–20 × 13–14 µm.

**FIGURE 3:**
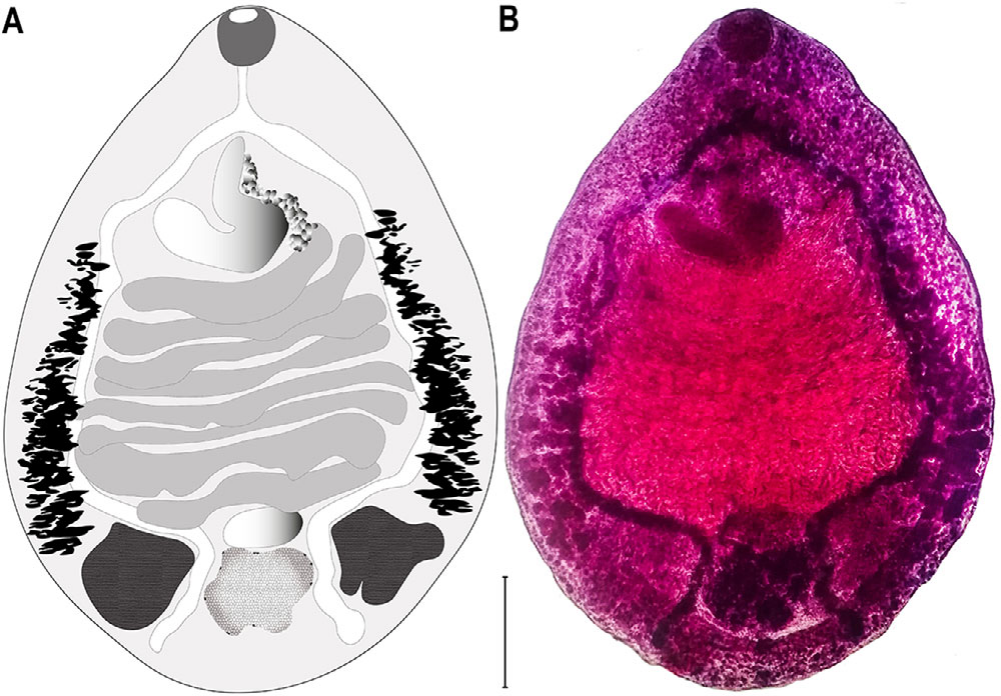
*Paramonostomum alveatum*, (A) Dorsal surface, (B) Light micrograph. Scale bar: 100 µm.

***Paramonostomum alveatum* Lühe, 1909** (Figure 3)

Host: *Aythya ferina, Netta rufina*

Site of infection: Small intestines

Intensity of infection: 35, 4

Locality: Bafra, Samsun, (41°36′N 36°05′E), Black Sea Region, Türkiye

Adults (based on ten specimens):

The body is ovoid, measuring 803–840 × 590–620 µm, with a length‐to‐width ratio of 1.36. Ventral glands are absent. The oral sucker is subterminal and circular, measuring 58–62 × 62–65 µm. A ventral sucker, prepharynx, and pharynx are absent.

The testes are slightly lobed and located in the posterior region of the body, posterior to the vitelline glands. The left testis measures 145–155 × 140–147 µm, while the right testis measures 144–150 × 120–140 µm. The cirrus sac is 234–240 µm in length, and the genital pore is positioned posterior to the intestinal bifurcation.

The ovary is oval and slightly lobed, located between the testes in the posterior part of the body, and measures 94–97 × 110–115 µm. The vitelline glands extend from the mid‐body to the level of the testes. The uterus is distinct and forms 9–10 transverse uterine loops. Eggs measure 19–21 × 10–11 µm.

**FIGURE 4:**
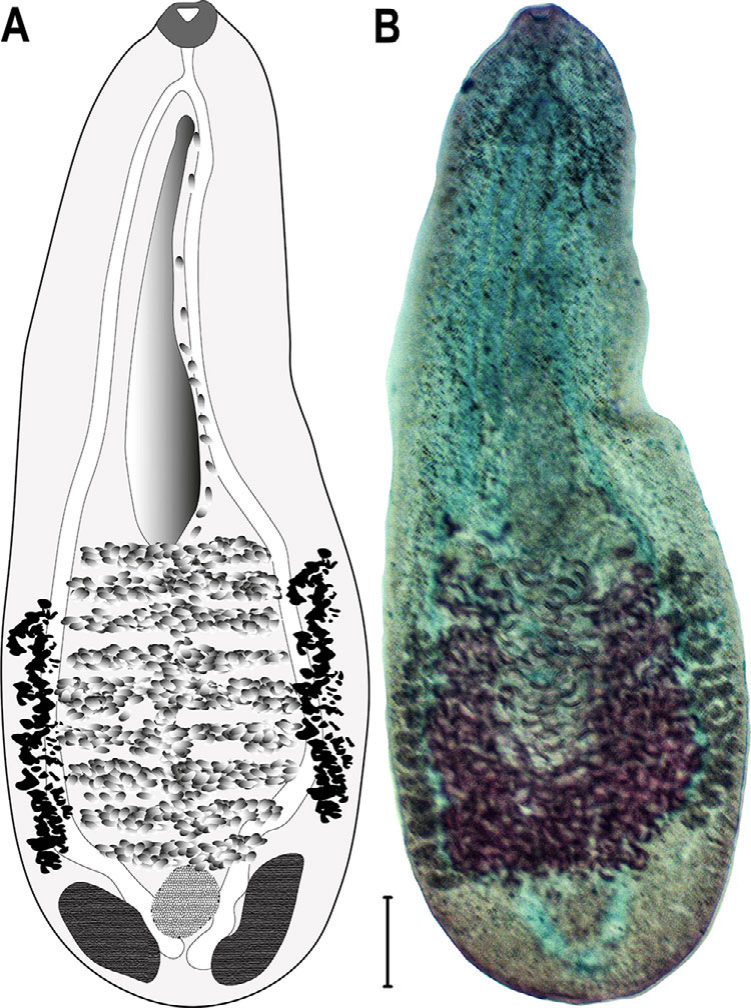
*Paramonostomum alveoelongatum*, (A) Dorsal surface, (B) Light micrograph. Scale bar: 100 µm.

***Paramonostomum alveoelongatum* Filimonova, 1971** (Figure 4)

Host: *Aythya ferina*

Site of infection: Small intestines

Intensity of infection: 3

Locality: Bafra, Samsun, (41°36′N 36°05′E), Black Sea Region, Türkiye

Adults (based on three specimens):

The body is slender, elongated, and ellipsoidal, measuring 775–785 µm in length and 180–185 µm in width, with a length‐to‐width ratio of 4.30. The ventral surface lacks glands. The oral sucker is subterminal and circular, measuring 42–45 × 48–50 µm. A ventral sucker, prepharynx, and pharynx are absent.

The testes are lobed and located in the posterior region of the body, posterior to the vitelline glands. The left testis measures 64–67 × 26–27 µm, whereas the right testis measures 62–65 × 30–34 µm. The cirrus measures 236–237 µm in length, and the genital pore is positioned posterior to the bifurcation of the cecum.

The ovary is oval and slightly lobed, located between the testes in the posterior part of the body, and measures 37–39 × 32–33 µm. The vitelline glands begin at the mid‐body level and extend posteriorly to the level of the testes. The uterus is evident and forms nine transverse uterine loops. Eggs measure 20–21 × 11–12 µm.

**FIGURE 5:**
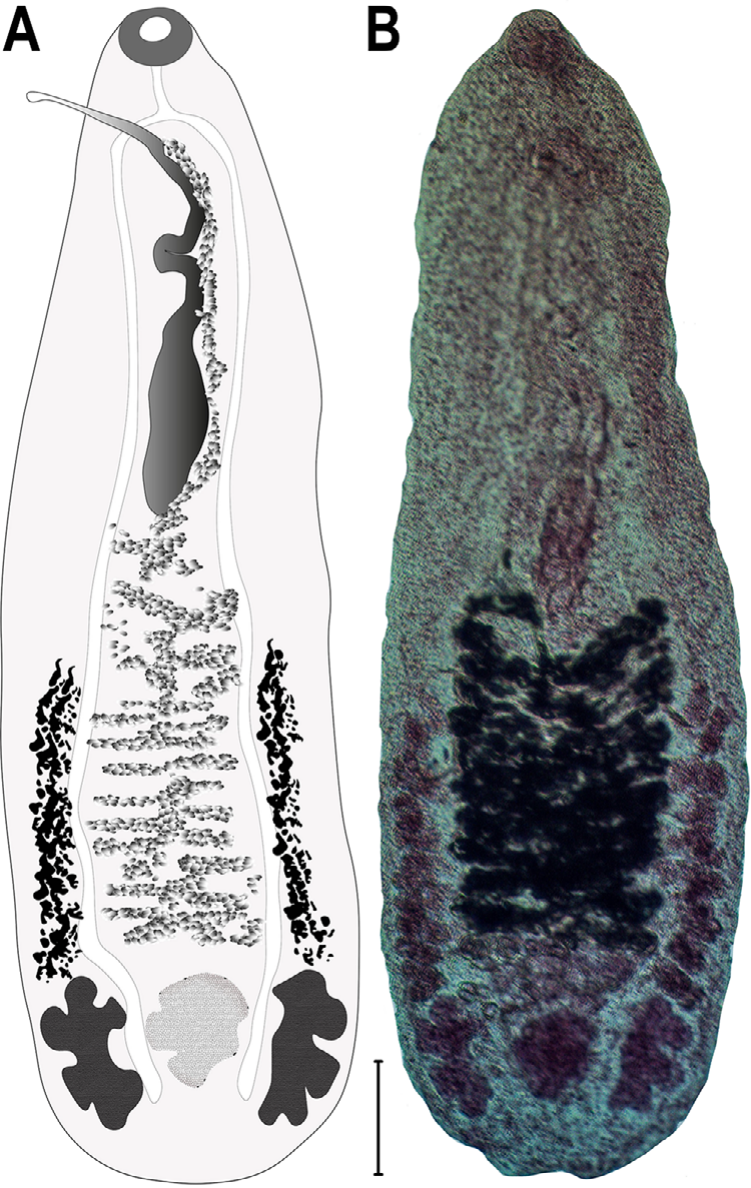
*Paramonostomum bucephale*, (A) Dorsal surface, (B) Light micrograph. Scale bar: 100 µm.

***Paramonostomum bucephalae* Yamaguti, 1935** (Figure 5)

Host: *Netta rufina*

Site of infection: Small intestines

Intensity of infection: 3

Locality: Bafra, Samsun, (41°36′N 36°05′E), Black Sea Region, Türkiye

Adults (based on three specimens):

The body is slender, elongated, and ellipsoidal, measuring 1.3–1.35 mm in length and 494–497 µm in width, with a length‐to‐width ratio of 2.78. The ventral surface lacks glands. The oral sucker is subterminal and circular, measuring 52–55 × 66–67 µm. A ventral sucker, prepharynx, and pharynx are absent.

The testes are slightly lobed and located in the posterior region of the body, posterior to the vitelline glands. The left testis measures 197–200 × 93–107 µm, whereas the right testis measures 150–155 × 83–87 µm. The cirrus measures 210–215 µm in length, and the genital pore is situated posterior to the bifurcation of the cecum.

The ovary is oval and slightly lobed, located between the testes in the posterior part of the body, and measures 80–85 × 111–115 µm. The vitelline glands begin at the mid‐body level and extend posteriorly to the level of the testes. The uterus is prominent and forms 15 transverse uterine loops. Eggs measure 19–20 × 10–11 µm

**FIGURE 6:**
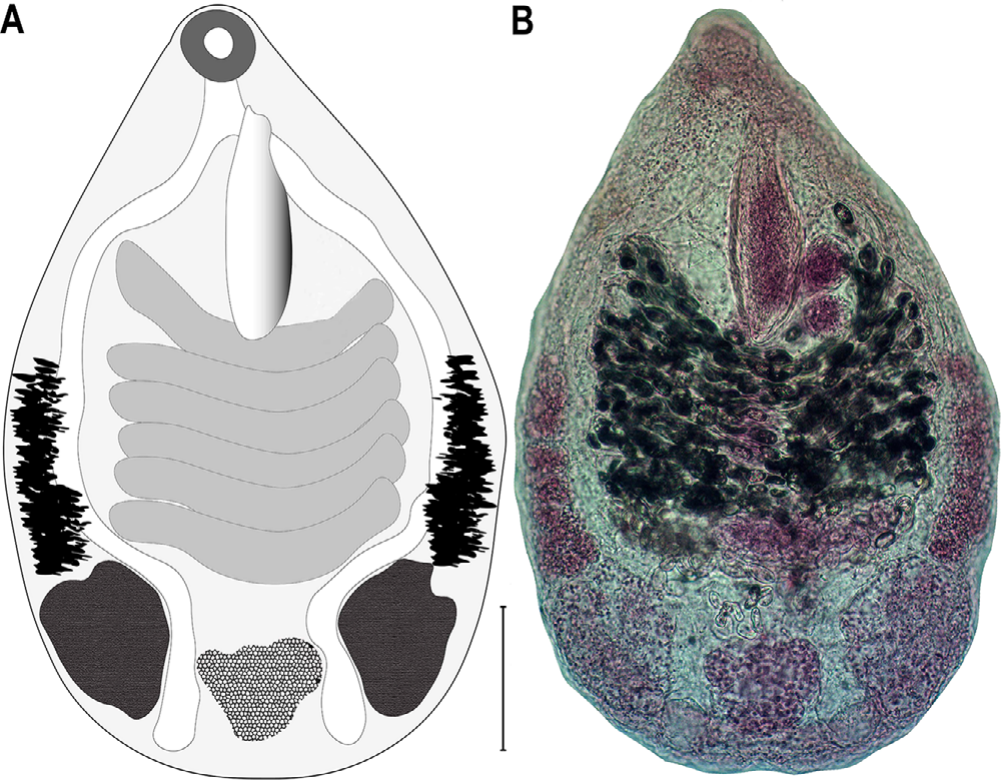
*Paramonostomum pseudoalveatum*, (A) Dorsal surface, (B) Light micrograph. Scale bar: 100 µm.

***Paramonostomum pseudoalveatum* Price, 1931** (Figure 6)

Host: *Netta rufina*

Site of infection: Small intestines

Intensity of infection: 3

Locality: Bafra, Samsun, (41°36′N 36°05′E), Black Sea Region, Türkiye

Adults (based on three specimens):

The body is oval to rounded, measuring 538–542 µm in length and 330–335 µm in width, with a length‐to‐width ratio of 1.63. The tegument is devoid of spines, and the ventral surface lacks glands. The oral sucker is subterminal and circular, measuring 98–102 × 109–111 µm. A ventral sucker, prepharynx, and pharynx are absent.

The testes are slightly lobed and located in the posterior region of the body, posterior to the vitelline glands. The left testis measures 110–112 × 78–83 µm, whereas the right testis measures 104–107 × 84–86 µm. The cirrus measures 160–165 µm in length, and the genital pore is situated anterior to the bifurcation of the cecum.

The ovary is oval and slightly lobed, located between the testes in the posterior part of the body, and measures 45–47 × 68–71 µm. The vitelline glands begin at the mid‐body level and extend posteriorly to the level of the testes. The uterus is evident and forms six transverse uterine loops. Eggs measure 17–18 × 10–11 µm.

## 4 Discussion

The morphological findings obtained in the present study are discussed below in the context of previous descriptions and geographical records. The genus *Notocotylus* within the family Notocotylidae is characterized by the presence of ventral glands arranged in longitudinal rows, the number of which varies among species (Table 2). In *Notocotylus attenuatus*, the number of ventral glands has been reported to differ considerably between studies. For example, descriptions of the Indian fauna indicate the presence of 18 glands in each row (Mehra 1980), whereas investigations on waterfowl trematodes have reported 14–19 glands in the lateral rows and 13–18 glands in the median row (McDonald 1981). In addition, ultrastructural observations using electron microscopy documented 14 glands in each row (Radlett 1980). In the present study, specimens of *N. attenuatus* recovered from *Rallus aquaticus* and *Netta rufina* consistently exhibited 14 glands in each ventral row. This observed variation in gland number is most likely attributable to differences related to host species and geographical origin.

**TABLE 2:** Comparative measurements of notocotylid trematodes (mm).

	*Notocotylus attenuatus* Rudolphi, 1809			*Notocotylus skrjabini* Ablasov, 1953	
Reference	Yamaguti (1934)	Skryabin (1953)	Present study	Ablasov (1953); Skryabin (1953)’ Filimonova (1985)	Present study
Body size	4.1–4.2 × 0.76	2.0–5.42 × 0,65–1.4	2.0–2.2 × 0.52–0.54	2.93–3.65 × 1.89–2.02	1.7–1.8 × 1.1–1.15
Oral sucker	0.13–0.15×0.15–0.16	0.105–0.27×0.14–0.21	0.095–0.11 × 0.104–0.115	0.22–0.25 × 0.23–0.26	0.149–0.155 × 0.17–0.175
Ventral glands	15–16 laterale, 14–15 median	13–17 laterale, 14–15 median	13–17 laterale, 14–15 median	5 laterale, 5 median	5 laterale, 5 median
Left testis	0.58–0.65×0.18–0.33	—	0.31–0.35 × 0.10–0.12	0,67–0,83 × 0,53–0,60	0.327–330 × 0.235–0.240
Right testis	0.58–0.65×0.18–0.33	—	0.46–0.5 × 0.11–0.15	0,67–0,83 × 0,53–0,60	0.328–330 × 0.217–0.216
Cirrus sac	—	0.77–1.96	0.710‐0.75	0.217–0.282 × 0.090–0.102	0.328–0.330
Ovary	0.18–0.28×0.25	—	0.27–0.28 × 0.11–0.12	0,33–0,38 × 0,30–0,31	0.195–0.20 × 0.139–0.142
Egg	0.017–0.020 × 0.09–0.0115	0.018–0.021 × 0.012	0.019–0.020 × 0.011–0.012	0.021–0.024 × 0.013	0.019–0.020 × 0.013–0.014

	*Paramonostomum alveatum* Lühe, 1909			*Paramonostomum alveoelongatum* Filimonova, 1971	
Reference	Skryabin (1953)	Lühe (1909); Stunkard (1967)	Present study	Filimonova (1985); McDonald (1981)	Present study
Body size	0.78–0.9 × 0.50–0.56	0.50–1.0 × 0.40–0.7	0.80–0.84 × 0.59–0.62	0.784–1.075 × 0.358–0.448	0.775–0.785 × 0.18–0.185
Oral sucker	0.059–0.115	0.05–0.09	0.058–0.062 × 0.062–0.065	0.056–0.079	0.042–0.045 × 0.048–0.050
Transverse uterus loops	—	10–12	9–10	9–11	9
Left testis	0.118‐0.147	0.12–0.18 × 0.10–0.14	0.145–155 × 0.14–0.147	0.125–0.200 × 0.086‐0.125	0.064–0.067 × 0.026–0.027
Right testis	0.118‐0.147	0.12–0.18 × 0.10–0.14	0.144–150 × 0.12–0.14	0.125–0.200 × 0.086–0.125	0.062–0.065 × 0.030–0.034
Cirrus sac	0.24 × 0.118	0.16–0.24 × 0.10–0.13	0.234–0.24	0.279–0.296	0.236–0.237
Ovary	0.084‐0.167	0.08‐0.15	0.094–0.097 × 0.110–0.115	0.086–0.103 × 0.090–0.108	0.037–0.039 × 0.032–0.033
Egg	0.021 × 0.011	0.019–0.021 × 0.010	0.019–0.021 × 0.010–0.011	0.022–0,025 × 0.011	0.020–0.021 × 0.011–0.012

	*Paramonostomum bucephalae* Yamaguti, 1935		*Paramonostomum pseudoalveatum* Price, 1931		
Reference	Skryabin (1953)	Present study	Price (1931)	Drago et al. (2007)	Present study
Body size	1.9–4.5 × 0.5–1.08	1.3–1.35 × 0.494–0.497	0.387–0.496 × 0.310–0.341	0.365–0.547 × 0.221–0.403	0.538–0.542 × 0.330–0.335
Oral sucker	0.078–0.111 × 0.09–0.138	0.052–0.055 × 0.066–0.067	0.038–0.052	0.034–0.053 × 0.028–0.053	0.098–0.102 × 0.109–0.111
Transverse uterus loops	—	15	—	4–7	6
Left testis	0.25–0,7 × 0.15–0.4	197–200 × 93–107	0.113–120 × 0.053–0.057	0.087–0.121 × 0.048–0.078	0.110–0.112 × 0.078–0.083
Right testis	0.25–0.7 × 0.15–0.4	150–155 × 83–87	0.113–120 × 0.053–0.057	0.078–0.111 × 0.048–0.078	0.104–0.107 × 0.084–0.086
Cirrus sac	0.65–1.68 × 0.06–0.16	210–215	0.074–0.097 × 0.037–0.060	0.090–0.145 × 0.031–0.050	0.160–0.165
Ovary	0.2‐0.25 × 0.16‐0.3	80–85 × 111–115	0.075–0.090 × 0.067–0.097	0.044–0.107 × 0.037–0.097	0.045–0.047 × 0.068–0.071
Egg	0.018–0.021 × 0.010–0.013	0.019–0.020 × 0.010–0.011	0.018–0.020 × 0.010	—	0.017–0.018 × 0.010–0.011

*Notocotylus skrjabini* has been regarded as morphologically similar to *N. breviserialis*, and some authors have suggested that these taxa may represent synonyms (Diaz et al. 2020). However, a key distinguishing feature between the two species is the number of ventral glands: *N. breviserialis* possesses four glands in each lateral row, whereas *N. skrjabini* is characterized by five glands in each lateral row (McDonald 1981, Boyce et al. 2012, Muniz‐Pereira and Amato 1995). Another morphologically comparable species is *N. lianhuaensis*, originally described from China. Although *N. lianhuaensis* resembles *N. skrjabini* in having three rows of ventral glands, it differs by having four glands in each lateral row, 6–7 median glands, deeply lobed testes, and a lobed ovary (Boyce et al. 2012, Li 1988). These morphological distinctions support the identification of the present specimens as *N. skrjabini*.

According to Bagnato et al. (Bagnato et al. 2022), species of the genus *Paramonostomum* can be grouped into four morphological categories, oval, pyriform, elongate, and overlong, based primarily on body shape and size (Table 2). Within this framework, *P. alveatum* belongs to the oval group. Among oval species, *P. alveatum* is markedly larger in body dimensions than *P. parvum*, *P. pseudoalveatum*, *P. anatis*, and *P. brantae*, yet smaller than *P. bursae*, *P. philippinensis*, *P. orientalis*, and *P. fulicae* (Bagnato et al. 2022, McDonald 1981). *Paramonostomum pseudoalveatum*, also classified within the oval group, is considerably smaller than *P. bursae*, *P. philippinensis*, *P. orientalis*, and *P. fulicae*. Furthermore, the position of the genital pore provides an additional diagnostic feature within this group: in *P. parvum* and *P. brantae*, the genital pore is located posterior to the intestinal bifurcation, whereas in *P. anatis* it is situated anterior to the bifurcation region (Bagnato et al. 2022, McDonald 1981).

In the present study, *P. alveoelongatum* and *P. bucephalae* were assigned to the elongate morphological group. *Paramonostomum alveoelongatum* is distinguished from other elongate species, such as *P. caeci*, *P. deseado*, and *P. actitidis*, by its relatively larger body dimensions and by the position of the genital pore precisely at the level of the intestinal bifurcation. Similarly, *P. bucephalae* differs from *P. musculus* and *P. histrionici* by its larger body size and by the genital pore being located slightly posterior to the bifurcation region (Bagnato et al. 2022, Karataş et al. 2021). In addition to these features, the number of transverse uterine loops represents an important taxonomic character. Within the oval group, *P. alveatum* typically exhibits 6–11 uterine loops, whereas *P. pseudoalveatum* shows 5–6 loops. In the elongate group, *P. alveoelongatum* possesses 9–11 uterine loops, while *P. bucephalae* is characterized by 15–16 loops (McDonald 1981).

*Paramonostomum bucephalae* was originally described from *Bucephala clangula* (Yamaguti 1935). Subsequent studies reported this species in several avian hosts from Japan, including *Anas clypeata*, *Nyroca marila*, and *Tadorna tadorna* (Skryabin 1953). It was later recorded from museum specimens of *Corvus frugilegus* in Hungary (Matskasi 1973). *Paramonostomum alveoelongatum* has previously been reported from Russia (McDonald 1981). In North America, *N. attenuatus* was first documented in *Anas crecca* in 1981 (Canaris et al. 1981) and was later reported from multiple waterfowl species in the United States and Mexico (Farias and Canaris 1986). In Bulgaria, *P. bucephalae* was identified in *Podiceps nigricollis* (Kostadinova et al. 1988), while *P. alveatum* was recorded from various bird species in Iraq (Farahnak et al. 2004). A Serbian study documented a prevalence of 22.88% for *N. attenuatus* in *Fulica atra* (Kulišić et al. 2004).

Between 1957 and 2000, studies conducted in the Czech Republic and Slovakia reported *N. attenuatus* in a wide range of avian hosts, including *Anas platyrhynchos*, *A. querquedula*, *A. strepera*, *Anser anser*, *A. fabalis*, *Aythya ferina*, *A. fuligula*, *Fulica atra*, *Melanitta fusca*, *Netta rufina*, *Phasianus colchicus*, *Podiceps cristatus*, *Somateria mollissima*, *Tachybaptus ruficollis*, and *Tringa ochropus* (Sitko et al. 2006). In the same region, *P. alveatum* was reported from *A. penelope*, *A. fabalis*, *Clangula hyemalis*, and *Somateria mollissima*, while *P. elongatum* and *P. parvum* were observed in *Cygnus* in Slovakia (Sitko et al. 2006).

In Poland, surveys conducted between 2001 and 2006 recorded *N. attenuatus* and *P. alveatum* in several waterfowl species, including *Anas strepera*, *A. crecca*, *A. platyrhynchos*, *Aythya marila*, *A. fuligula*, *Melanitta nigra*, *M. fusca*, and *Mergus merganser* (Kavetska et al. 2008). Later studies reported infection rates of *N. attenuatus* reaching 20.2% in *Aythya fuligula* and 22.2% in *Aythya marila* in Poland (Rząd et al. 2013). In Iran, *N. attenuatus* was documented with a prevalence of 26.4% in *A. crecca* (Youssefi et al. 2014). In Türkiye, Hügül reported infection rates of *N. attenuatus* of 16.66% in *A. ferina*, 100% in *A. fuligula*, and 50% in *A. strepera* at Lake Çavuşcu (Güçlü and Hüğül 2020). In the same study, *P. alveatum* was detected in *A. ferina* with a prevalence of 33.33%, while *P. pacifera* was recorded from *Fulica atra*.

The present study increases the number of notocotylid trematode species recorded from wild birds in Türkiye from three to eight. Previously reported species included *Notocotylus attenuatus*, *Paramonostomum alveatum*, and *Paramonostomum pacifera*. Newly recorded species are *N. skrjabini*, *P. alveoelongatum*, *P. bucephalae*, and *P. pseudoalveatum*. The detection of these species highlights the importance of continued parasitological surveys in Türkiye and suggests that the diversity of the Notocotylidae fauna in the region has been underestimated.

The distribution and prevalence of notocotylid trematodes in Türkiye are likely influenced by multiple factors, including climatic conditions, habitat availability, the presence of suitable intermediate hosts such as aquatic snails, and the migratory behaviour of birds. The Kızılırmak Delta, a Ramsar‐designated wetland where the present study was conducted, provides favourable ecological conditions for both avian hosts and intermediate hosts, thereby facilitating the transmission of Notocotylidae trematodes.

## 5 Conclusion

This study provides a comprehensive morphological survey of notocotylid trematodes infecting wild birds in Türkiye and significantly expands the current knowledge of the family Notocotylidae in the region. A total of six species belonging to the genera *Notocotylus* and *Paramonostomum* were identified, four of which (*Notocotylus skrjabini*, *Paramonostomum alveoelongatum*, *P. bucephalae*, and *P. pseudoalveatum*) are reported from Türkiye for the first time.

Detailed morphological examinations confirmed the taxonomic identity of all recovered species and allowed reliable differentiation based on key diagnostic characters, including ventral gland arrangement, body proportions, genital pore position, and the number of transverse uterine loops. These findings highlight the importance of careful morphological analysis in notocotylid systematics, particularly in regions where parasitological data remain limited.

The results also underscore the role of wetlands such as the Kızılırmak Delta as important habitats for the transmission and maintenance of notocotylid trematodes, owing to the presence of suitable avian hosts and aquatic intermediate hosts. Continued parasitological surveys integrating detailed morphological approaches are essential to further elucidate the diversity, host associations, and geographical distribution of Notocotylidae in Türkiye.

## Author Contributions

**Mehmet Öztürk**: Conceptualization, data curation, formal analysis, funding acquisition, investigation, methodology, project administration, software, visualization, writing – original draft, writing – review and editing; **Şinasi Umur**: Conceptualization, data curation, formal analysis, funding acquisition, resources, software, supervision, validation, writing – original draft, writing – review and editing.

## Funding

This study is funded by TÜBİTAK (Türkiye Bilimsel ve Teknolojik Araştırma Kurumu) under project number 222O228

## Ethics Statement

The authors confirm that the journal's ethical policies, as noted on the journal's author guidelines page, have been adhered to. Specific Ethics and Welfare Committee approval was not required, as naturally dead material was used.

## Conflicts of Interest

The authors declare no conflicts of interest.

## Consent to Participate

All authors confirm their participation in the study.

## Data Availability

Data are available from the corresponding author upon reasonable request.
